# Relationships between test positivity rate, total laboratory confirmed cases of malaria, and malaria incidence in high burden settings of Uganda: an ecological analysis

**DOI:** 10.1186/s12936-021-03584-7

**Published:** 2021-01-13

**Authors:** Jaffer Okiring, Adrienne Epstein, Jane F. Namuganga, Victor Kamya, Asadu Sserwanga, James Kapisi, Chris Ebong, Simon P. Kigozi, Arthur Mpimbaza, Humphrey Wanzira, Jessica Briggs, Moses R. Kamya, Joaniter I. Nankabirwa, Grant Dorsey

**Affiliations:** 1 Clinical Epidemiology Unit, School of Medicine, Makerere University College of Health Sciences, Kampala, Uganda; 2 Department of Epidemiology and Biostatistics, University of California, San Francisco, USA; 3 Infectious Diseases Research Collaboration, 2C Nakasero Hill Road, Kampala, Uganda; 4 Pilgrim Africa, Kampala, Uganda; 5 Department of Medicine, University of California, San Francisco, USA

**Keywords:** Malaria, Surveillance, Metrics, Test positivity rate, Cases, Incidence

## Abstract

**Background:**

Malaria surveillance is critical for monitoring changes in malaria morbidity over time. National Malaria Control Programmes often rely on surrogate measures of malaria incidence, including the test positivity rate (TPR) and total laboratory confirmed cases of malaria (TCM), to monitor trends in malaria morbidity. However, there are limited data on the accuracy of TPR and TCM for predicting temporal changes in malaria incidence, especially in high burden settings.

**Methods:**

This study leveraged data from 5 malaria reference centres (MRCs) located in high burden settings over a 15-month period from November 2018 through January 2020 as part of an enhanced health facility-based surveillance system established in Uganda. Individual level data were collected from all outpatients including demographics, laboratory test results, and village of residence. Estimates of malaria incidence were derived from catchment areas around the MRCs. Temporal relationships between monthly aggregate measures of TPR and TCM relative to estimates of malaria incidence were examined using linear and exponential regression models.

**Results:**

A total of 149,739 outpatient visits to the 5 MRCs were recorded. Overall, malaria was suspected in 73.4% of visits, 99.1% of patients with suspected malaria received a diagnostic test, and 69.7% of those tested for malaria were positive. Temporal correlations between monthly measures of TPR and malaria incidence using linear and exponential regression models were relatively poor, with small changes in TPR frequently associated with large changes in malaria incidence. Linear regression models of temporal changes in TCM provided the most parsimonious and accurate predictor of changes in malaria incidence, with adjusted R^2^ values ranging from 0.81 to 0.98 across the 5 MRCs. However, the slope of the regression lines indicating the change in malaria incidence per unit change in TCM varied from 0.57 to 2.13 across the 5 MRCs, and when combining data across all 5 sites, the R^2^ value reduced to 0.38.

**Conclusions:**

In high malaria burden areas of Uganda, site-specific temporal changes in TCM had a strong linear relationship with malaria incidence and were a more useful metric than TPR. However, caution should be taken when comparing changes in TCM across sites.

## Background

Malaria surveillance is considered a core intervention and critical for the purposes of monitoring and evaluation [1–3]. However, for many countries in sub-Saharan Africa, malaria surveillance systems are limited in their ability to accurately monitor trends in malaria morbidity. The most widely available source of routine malaria surveillance data come from national health management information systems (HMIS). HMIS data typically includes aggregate numbers of patients tested for malaria and diagnosed with malaria. The “gold standard” metric for malaria morbidity is malaria incidence, defined as the number of cases of laboratory confirmed malaria per unit time divided by the size of the population at risk [4]. Although the quality of HMIS data has improved over the last decade in most countries in sub-Saharan Africa due to expanded diagnostics and a reliance on laboratory confirmed cases of malaria, it is not possible to routinely estimate malaria incidence because of lack of information on where patients reside and undefined catchment populations around the health facilities. Therefore, the monitoring of temporal and geographic trends in malaria morbidity using HMIS data typically relies on surrogate measures of malaria incidence such as the test positivity rate (TPR) or total laboratory confirmed cases of malaria (TCM).

The TPR is defined as the number of laboratory confirmed cases of malaria per 100 patients tested for malaria. Advantages of the TPR include that it is relatively easy to measure and is not dependent on the numbers of patients coming to a health facility or undergoing diagnostic testing, assuming that there is no differential bias in who accesses care or undergoes testing at the facility. However, temporal trends in the TPR may be susceptible to bias due to changes in diagnostic testing, health care-seeking behaviour, and the incidence of non-malarial febrile illnesses [5]. In addition, TPR has a non-linear relationship with malaria incidence and in high endemic settings, small changes in TPR can be associated with large changes in malaria incidence [6, 7]. More still, TPR is a proportion, commonly used as a qualitative measure as it is difficult to translate changes in TPR into meaningful quantitative measures needed to allocate resources and assess impact. TCM simply represents the numerator of the TPR and is also relatively easy to measure [8]. Unlike the TPR, the TCM is not constrained between 0 and 100. However, this metric lacks a clear denominator and is highly dependent on diagnostic practices at a health facility, changes in the catchment area or catchment population, and any factors that may impact care-seeking behaviours, such as poor weather, drug stock-outs, access to other health facilities, or community-based programmes [9, 10].

In Uganda, an enhanced health facility-based malaria surveillance system was established to provide high quality data at sentinel sites around the country referred to as Malaria Reference Centers (MRCs) [11]. At these MRCs, individual patient level data is collected and resources are provided to maximize laboratory testing of all patients with suspected malaria. More recently, data on village of residence has been captured and catchment areas around the MRCs identified, allowing for the generation of estimates of malaria incidence. In this study, temporal relationships between TPR and TCM relative to malaria incidence estimates were examined at five MRCs over a 15-month period in areas where the burden of malaria is high.

## Methods

### Establishment of health-facility based malaria surveillance system

Data for this study come from the Uganda Malaria Surveillance Project (UMSP). UMSP in collaboration with the Uganda National Malaria Control Division (NMCD) established a health facility-based malaria surveillance system at several MRCs beginning in 2006. MRCs are high volume level III/IV public health facilities that generally see between 1000 and 3000 outpatients per month and have functioning laboratories. At each MRC, individual-level data from standardized HMIS registers for all patients presenting to the outpatient departments are entered into an Access database by on-site data officers. Primary data captured comes from the HMIS 031 standardized form (Additional file 1: Appendix 1) and includes village of residence, age, gender, type of malaria test done (rapid diagnostic test (RDT) or microscopy), and malaria diagnostic test results. The research team supports the sites with training, site support supervision, and buffer stock of laboratory supplies/consumables. Full-time regional surveillance assistants are based around the country; each supervising 8–10 MRCs. Site support supervision is conducted on a regular basis to provide refresher training and onsite mentorship on malaria case management, malaria microscopy, conduct data use meetings and provide feedback on performance, and to conduct laboratory external quality control for malaria microscopy. Core team members are also responsible for generating periodic reports, communicating with Ministry of Health officials and other key stakeholders, and conducting data analyses.

**Fig. 1:**
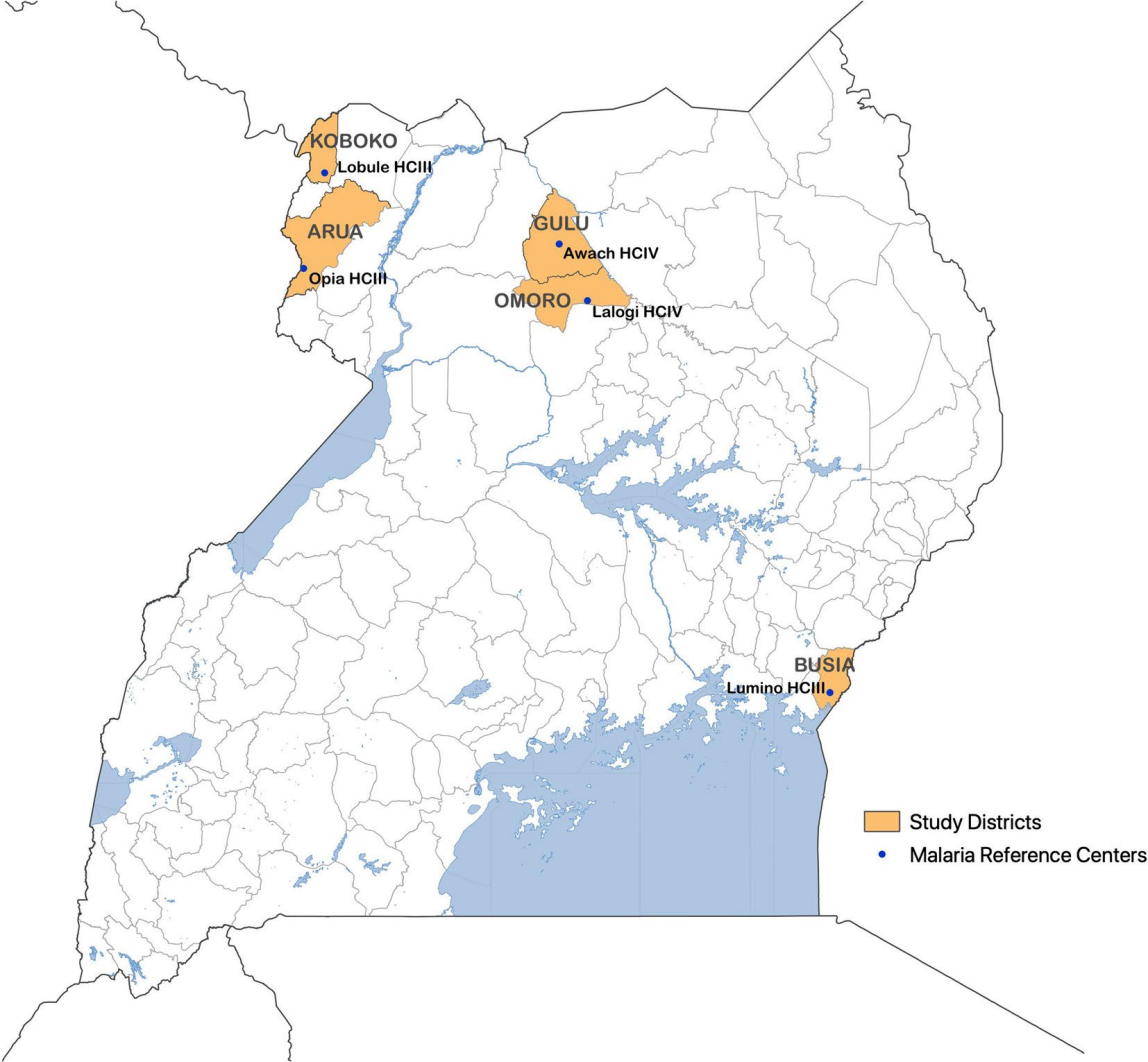
Map of Uganda showing the study districts and malaria reference centres

This study included data from five MRCs which met the following criteria: (1) location in a high malaria burden area where indoor residual spraying of insecticide (IRS) is not being implemented, and (2) less than 5% missing data from November 2018 through January 2020 for each of the following variables; age (all patients), village of residence (all patients), and results for malaria diagnostic testing (among patients with suspected malaria). Suspected malaria was defined as all patients referred for malaria laboratory testing plus all patients not referred for a malaria laboratory test but given a clinical diagnosis of malaria. These facilities include Lobule health centre III in Koboko District, Opia health centre III in Arua District, Awach health centre IV in Gulu District, Lalogi health centre IV in Omoro District, and Lumino health centre III in Busia District (Fig. 1).

### Malaria metrics

TPR was defined as the proportion of all patients tested for malaria who tested positive. TCM was defined as the number of all patients who tested positive for malaria (numerator of the TPR). To generate estimates of malaria incidence, catchment areas were identified around the MRCs based on the assumption that the majority of patients within the catchment area who developed malaria would be captured by the surveillance system. Catchments areas included the village where the MRC is located and adjacent villages that met all of the following criteria: (1) did not contain another public health facility, (2) were in the sub-county where the MRC is located, (3) had a similar incidence of malaria as the village where the MRC is located, and (4) provided an estimated total catchment area population of at least 1400 persons. Village level population estimates were obtained from the AfriPop database and included a fixed population growth function [12]. Catchment areas around each MRC included between 1 and 5 villages (Additional file 2: Appendix 2). Estimates of malaria incidence were defined as the total number of laboratory confirmed cases of malaria from patients residing within the catchment area (adjusted for missing data on malaria test results and village of residence) per unit time divided by the population of the catchment area.

### Statistical analysis

Data were analysed using Stata version 14.1 (College Station, TX). Cumulative data for the characteristics of the study populations were summarized over the 15-month observation period (November 2018 to January 2020). Data were aggregated by monthly intervals for all analyses of longitudinal trends. The maximal fold changes were defined as the ratio of maximum monthly value divided by the minimum monthly value for each metric, and used to describe the within site variation in TPR, TCM and malaria incidence during the observation period. Temporal correlations between TPR and malaria incidence as well as between TCM and malaria incidence stratified by MRC were made using linear and exponential terms with goodness-of-fit between models compared using Akaike Information Criteria (AIC). Final models of temporal correlations between TCM and malaria incidence stratified by MRC were made using standard linear regression with model characteristics summarized as the slope (95% CI) and adjusted R-squared value. Temporal correlations between TCM and malaria incidence for all 5 sites combined were estimated using a linear regression model with a random effect for study site. Selected analyses were also restricted to only patients under 5 years of age.

## Results

### Characteristics of the study population

Over the 15 month study period there were a total of 149,739 outpatient visits, ranging from 20,671 to 40,445 visits across the five MRCs. Malaria was suspected in 73.4% of all outpatient visits, ranging from 58.7 to 91.9% across the five MRCs. Among patients with suspected malaria, 99.1% had a diagnostic test done and 96.6% of these were tested using a RDT (the remainder being tested using microscopy). Overall, 69.7% of those tested for malaria were positive, with TPRs ranging from 59.8 to 77.3% across the five MRCs (Table 1). Overall, 50.7% of all patients presenting to the outpatient departments of these five MRCs had a laboratory confirmed diagnosis of malaria, highlighting the predominant role of malaria on the burden of disease at these facilities. When considering only children less than 5 years of age, testing rates and use of RDTs were similar, however, the proportion of patients with suspected malaria and TPRs were slightly higher across all five MRCs.

**Table 1:** Characteristics of the study population from November 2018 through January 2020

Age group	Characteristic	MRC				
		Awach	Lalogi	Opia	Lumino	Lobule
All ages	Total visits to outpatient departments	40,445	38,549	20,671	26,343	23,731
	Visits with malaria suspected (% total visits)	23,739 (58.7)	24,273 (63.0)	17,432 (84.3)	22,669 (86.1)	21,819 (91.9)
	RDT or microscopy done (% suspected)	22,828 (96.2)	24,246 (99.9)	17,420 (99.9)	22,577 (99.6)	21,818 (100)
	Tested using RDT (% tested)	21,815 (95.6)	23,458 (96.7)	17,407 (99.9)	21,829 (96.7)	20,694 (94.8)
	Positive malaria test (% tested)	16,872 (73.9)	16,521 (68.1)	12,170 (69.9)	13,510 (59.8)	16,867 (77.3)
Age < 5 years	Total visits to outpatient departments	7561	8140	3717	5917	5300
	Visits with malaria suspected (% total visits)	5222 (69.1)	5778 (71.0)	3214 (86.5)	5503 (93.0)	5090 (96.0)
	RDT or microscopy done (% suspected)	5002 (95.8)	5772 (99.9)	3214 (100.0)	5476 (99.5)	5089 (100)
	Tested using RDT (% tested)	4777 (95.5)	5510 (95.5)	3212 (99.9)	5133 (93.7)	4339 (85.3)
	Positive malaria test (% tested)	3858 (77.1)	4428 (76.7)	2265 (70.5)	4058 (74.1)	4263 (83.8)

### Summary data on longitudinal measures of malaria morbidity

Descriptive statistics of monthly aggregate measures of malaria morbidity for each MRC are presented in Table 2. Between sites, median monthly TPR values ranged from 59.4% in Lumino to 76.4% in Lobule. Results were similar when median monthly TPR values were restricted to only patients from the catchment areas. Within sites, monthly TPR values varied from a maximal 1.3-fold change in Lobule to a 2.4-fold change in Opia. Compared to TPR values, there was greater variation in monthly TCM values and estimates of malaria incidence, both between and within sites. Between sites, median monthly TCM values ranged from 700 in Opia to 1131 in Lobule. Within site monthly TCM values varied from a maximal 3.0-fold change in Lumino to a 7.8-fold change in Opia. Between sites, median monthly estimates of malaria incidence ranged from 744 cases per 1000 person years from the catchment area around Lalogi to 1689 cases per 1000 person years from the catchment area around Opia. Within site monthly estimates of malaria incidence varied from a maximal 2.9-fold change in Lobule to an 8.1-fold change in Opia. Similar findings were seen when data were restricted to only children under 5 years of age.

**Table 2:** Summary data on longitudinal measures of malaria morbidity

Age group	Monthly metrics, median (range)	MRC				
		Awach	Lalogi	Opia	Lumino	Lobule
All ages	Test positivity rate (TPR) all patients	69.2% (60.5–87.0%)	67.0% (40.8–83.2%)	63.0% (35.6–85.9%)	59.4% (51.9–70.7%)	76.4% (67.1–84.1%)
	Test positivity rate (TPR) from catchment area	73.3% (61.3–86.9%)	69.1% (40.7–86.4%)	68.4% (40.8–87.9%)	62.0% (50.2–73.2%)	76.3% (71.1–88.1%)
	Total laboratory confirmed cases of malaria (TCM)	994 (612–1951)	1,030 (349–2465)	700 (210–1633)	776 (570–1724)	1,131 (534–1695)
	Estimated cases of malaria from catchment area	594 (398–1194)	370 (118–913)	307 (90–711)	124 (90–353)	178 (111–327)
	Proportion of TCM from catchment area	63.4% (56.2–70.9%)	39.2% (30.4–44.5%)	43.2% (34.3–52.5%)	16.0% (12.9–20.5%)	18.0% (13.2–20.8%)
	Estimated population of catchment area	5239 (5134–5347)	5919 (5801–6041)	2170 (2126–2214)	1487 (1457–1517)	2871 (2814–2930)
	Malaria incidence (MI) from catchment area*	1357 (921–2726)	744 (244–1846)	1689 (489–3946)	1010 (721–2879)	761 (473–1351)
	Maximal fold change in TPR (all patients)	1.4	2.0	2.4	1.4	1.3
	Maximal fold change in TCM (all patients)	3.2	7.1	7.8	3.0	3.2
	Maximal fold change in MI (catchment area only)	3.0	7.6	8.1	4.0	2.9
< 5 years	Test positivity rate (TPR) all patients	72.0% (57.6–90.7%)	75.1% (43.8–89.9%)	65.5% (32.4–91.3%)	72.2% (62.7–85.9%)	83.1% (70.7–89.3%)
	Test positivity rate (TPR) from catchment area	74.0% (55.3–88.2%)	77.6% (40.0–90.9%)	69.5% (26.5–90.0%)	67.9% (54.5–83.8%)	83.7% (67.2–91.1%)
	Total laboratory confirmed cases of malaria (TCM)	230 (129–449)	254 (98–730)	129 (39–332)	256 (156–544)	284 (159–422)
	Estimated cases of malaria from catchment area	139 (80–291)	97 (31–270)	64 (13–156)	34 (19–108)	51 (29–74)
	Proportion of TCM from catchment area	61.1% (47.6–65.4%)	42.9% (30.8–51.0%)	45.1% (32.5–60.6%)	13.6% (10.2–19.8%)	19.8% (13.8–24.6%)
	Estimated population of catchment area	975 (956–995)	1102 (1,080–1124)	404 (396–412)	277 (271–283)	535 (524–546)
	Malaria incidence (MI) from catchment area*	1681 (976–3559)	1047 (340–2931)	1905 (390–4660)	1448 (838–4718)	1161 (659–1642)
	Maximal fold change in TPR (all patients)	1.6	2.1	2.8	1.4	1.3
	Maximal fold change in TCM (all patients)	3.5	7.4	8.5	3.5	2.7
	Maximal fold change in MI (catchment area only)	3.6	8.6	11.9	5.6	2.5

^*^cases per 1000 person years

### Temporal trends and correlations between measures of malaria morbidity

**Fig. 2:**
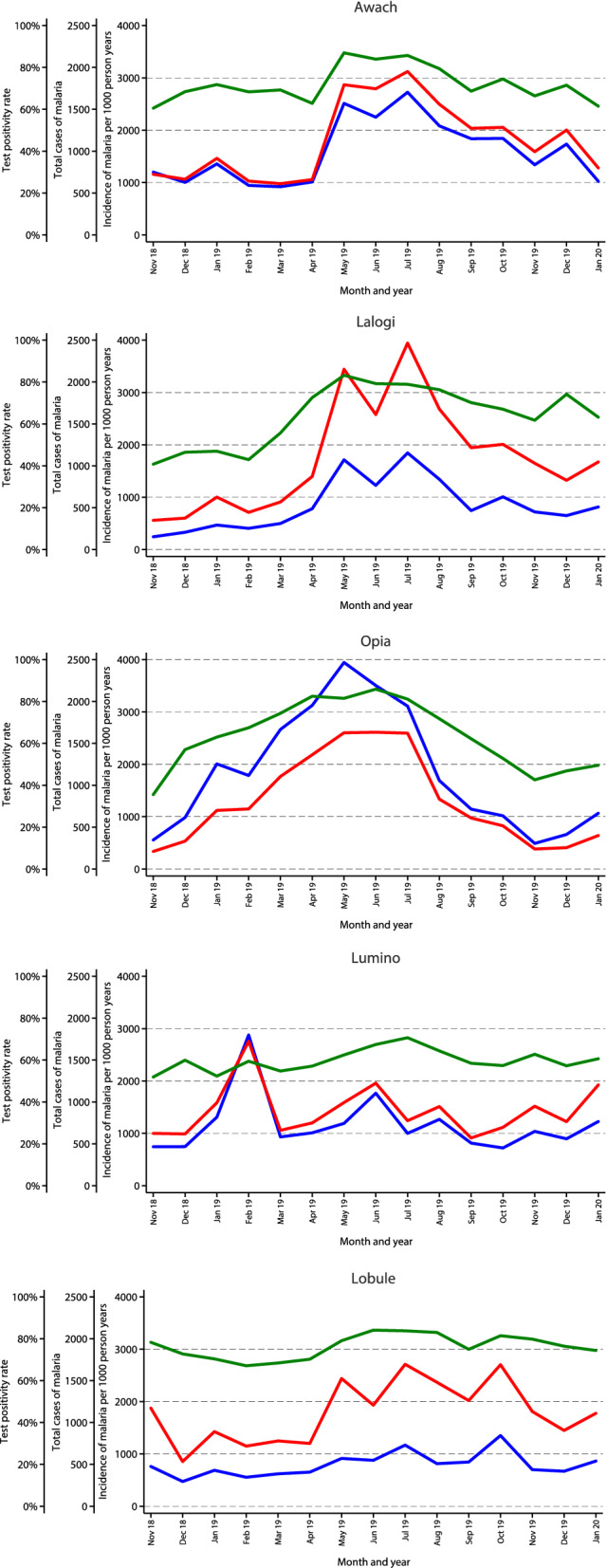
Temporal changes in monthly measures of malaria morbidity over the 15 month observation period for each MRC: TPR (green line), TCM (blue line), and malaria incidence (red line)

Temporal changes in monthly measures of malaria morbidity over the 15-month observation period for each MRC are presented in Fig. 2. A consistent temporal pattern was seen in the three metrics of malaria burden at each MRC, with peaks between April and August 2019 following the annual long rainy season that occurs in most of the country. Smaller peaks were seen at some sites following the shorter rainy season that occurs between November and January, with the exception of Lumino which had a large peak in TCM and malaria incidence in February 2019. Qualitatively all three metrics tracked relatively well together over time at all the sites, although monthly changes in TCM tracked better with malaria incidence compared to monthly changes in TPR.

**Fig. 3:**
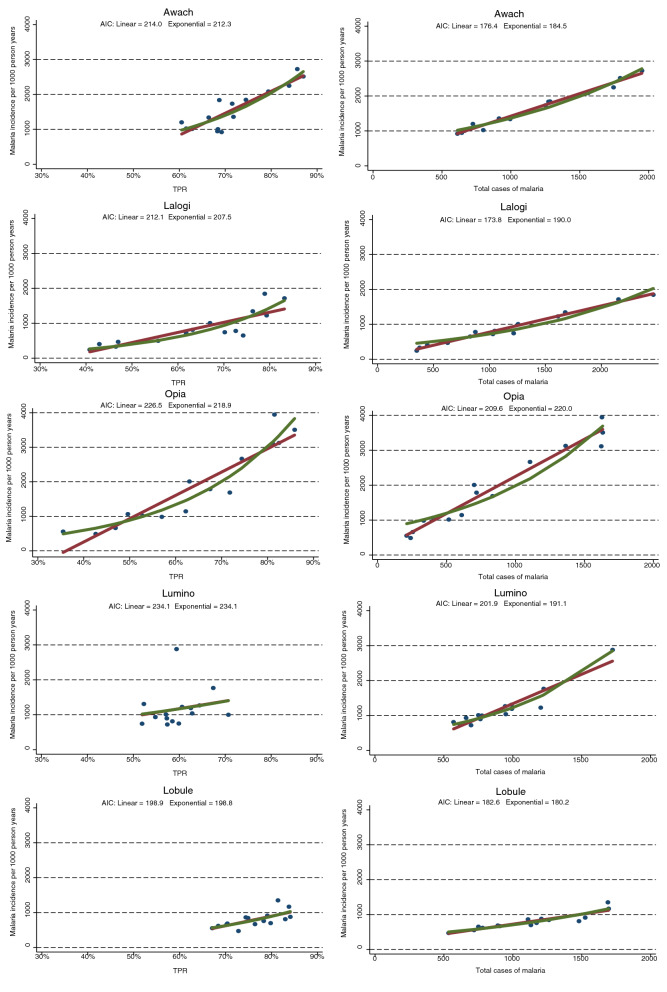
Linear (red line) and exponential (green line) models of the relationships between **a** TPR vs. malaria incidence, and **b** TCM and malaria incidence for each MRC. Blue dots represent observed values. AIC = Akaike Information Criteria

The assessment of temporal relationships between routinely available metrics of malaria morbidity, including TPR and TCM, with estimates of malaria incidence in the catchment areas around the MRCs are provided in Fig. 3. Linear correlations between TPR and malaria incidence were relatively poor, especially in Lumino and Lobule. Indeed, small changes in TPR were frequently associated with large changes in malaria incidence. The use of an exponential model improved model fit at 4 of the 5 sites, but only marginally. In contrast, linear correlations between TCM and malaria incidence were much stronger with improved model fit at all the sites when compared to either linear or exponential correlations between TPR and malaria incidence. Compared to linear correlations, exponential correlations between TCM and malaria inidence worsened model fit for 3 of the sites and was associated with only modest improved fit at 2 of the sites. In summary, linear regression models of temporal changes in TCM provided the most parsimonious and accurate predictor of changes in malaria incidence across the 5 high burden sites included in this study.

**Fig. 4:**
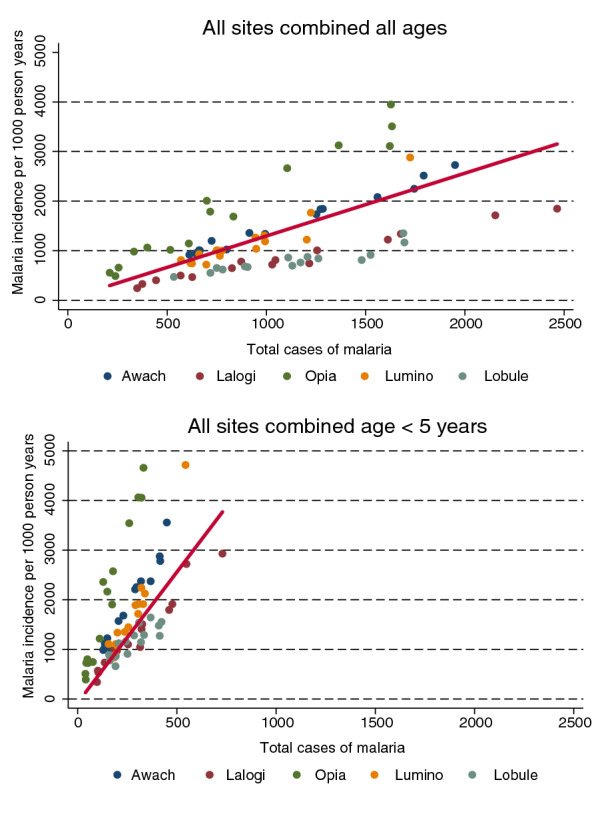
Linear (red line) model of the relationship between TCM and malaria incidence for all 5 MRCs combined stratified by **a** patients of all ages, and **b** only patients < 5 years of age. Colored dots represent observed values stratified by MRC

To further quantify the relationships between temporal changes in TCM and malaria incidence, the slope and adjusted R^2^ values for linear regression models for each site and all sites combined are presented in Table 3. Overall, TCM was an excellent predictor of malaria incidence for the individual sites with adjusted R^2^ values ranging from 0.81 to 0.98. Findings were similar when restricting the analysis to only children less than 5 years of age, although at one site (Lobule) the adjusted R^2^ value was only 0.68. In contrast, when combining data across all 5 sites, the R^2^ value reduced to 0.38 when considering all patients and 0.35 when only considering children less than 5 years of age (Table 3, Fig. 4). Furthermore, the slope of the regression lines indicating the change in malaria incidence per unit change in TCM varied across the sites. For example in Lobule a doubling in TCM was indicative of a 57% increase in malaria incidence (slope = 0.57), while in Opia and doubling in TCM was indicative of a 213% increase in malaria incidence (slope = 2.13). When considering only children under 5 years of age, the relative changes in malaria incidence per unit change in TCM were even greater with slopes ranging from 2.78 to 13.4 across the 5 sites.

**Table 3:** Linear regression models of Total laboratory confirmed cases of malaria as predictors of malaria incidence

MRC	All ages		Age < 5 years	
	Slope (95% CI)^a^	Adjusted R^2^	Slope (95% CI)^a^	Adjusted R^2^
Awach	1.29 (1.19–1.39)	0.98	7.03 (6.17–7.88)	0.96
Lalogi	0.75 (0.68–0.82)	0.98	3.99 (3.45–4.52)	0.95
Opia	2.13 (1.86–2.39)	0.95	13.4 (11.9–14.8)	0.97
Lumino	1.68 (1.32–2.04)	0.88	9.41 (7.89–10.9)	0.93
Lobule	0.57 (0.41–0.73)	0.81	2.78 (1.71–3.86)	0.68
All sites combined^b^	1.27 (0.78–1.75)	0.38	5.27 (3.42–7.13)	0.35

^a^ Change in incidence of malaria per 1000 person years / change in total laboratory confirmed cases of malaria

^b^ Random effects model (R^2^ unadjusted in models using random effects)

## Discussion

This study used data routinely collected at health facilities to generate two common metrics of malaria morbidity, TPR and TCM, and compared temporal relationships between these metrics with direct estimates of malaria incidence in 5 high burden areas of Uganda. In this setting, changes in TPR were poor predictors of changes in malaria incidence, with small changes in TPR often associated with large changes in malaria incidence. In contrast, site specific changes in TCM exhibited a strong linear relationship with changes in malaria incidence, suggesting this metric could provide a useful indicator of relative changes in malaria morbidity over time within sites. However, relationships between absolute changes in TCM and absolute changes in malaria incidence varied from site to site, limiting the ability to directly translate changes in TCM to changes in malaria incidence.

Malaria surveillance is essential to monitor trends over time and space and evaluate the impact of control interventions. In settings in which transmission remains relatively high, surveillance activities focused on measures of malaria morbidity provide the most useful data for analysis of trends, stratification, and planning of resource allocation [2, 13, 14]. In most high endemic countries, routine health information systems involving health facilities provide the only practical, continuous, and systematic source of data on malaria morbidity. However, the utility of routine data from health facilities may be limited by incomplete or inaccurate reporting, lack of diagnostic testing in patients with suspected malaria, and poor quality laboratory diagnostics. Despite these challenges, an increased emphasis on laboratory-based confirmation of malaria and widespread availability of RDTs has improved the quality and utility of routine health facility-based data [11, 15–17].

A strength of the current study was the use of high quality data from an enhanced malaria surveillance system at sentinel sites with a strong emphasis placed on complete reporting and laboratory confirmation for the diagnosis of malaria. Indeed, the fact that over 99% of patients with suspected malaria underwent diagnostic testing and over 96% of those tested had an RDT greatly reduced the potential for bias due to variations in these factors. Another strength of this study was the availability of estimates of malaria incidence from catchment areas around the health facilities. Malaria incidence provides the most direct measure of malaria burden and allows one to quantify cases over time relative to the size of the population at risk. The most accurate method of estimating malaria incidence involves prospective cohort studies, where all cases of malaria are captured from a defined study population [5, 18–20]. However, cohort studies require considerable resources and are rarely undertaken as part of routine surveillance programmes. In this study, a practical and low-cost method was used to estimate malaria incidence by improving the capture of routine data on the village of residence among patients presenting to the health facilities, mapping catchment areas around the facilities, and estimating the population of these catchment areas. Indeed, although village of residence is included on the HMIS 031 standardized form, under routine circumstances this is rarely filled out and when it is filled out, fraught with inaccuracies and no way of linking this information to any meaningful population level data. Indeed, one of the key (and pain-staking) aspects of the “enhanced” surveillance system used in this study was training the staff at the MRCs to accurately fill out the village of residence, creating a novel coding system for entering this into an electronic database, and creating maps and shapefiles that would allow the linking of malaria cases to catchment areas and estimating the populations of these catchment areas. Generating direct estimates of malaria incidence provided a means of assessing the accuracy of surrogate measures of malaria morbidity, including TPR and TCM, in predicting changes over time.

TPR, defined as the number of laboratory confirmed cases per 100 suspected cases examined, has been used to define levels of endemicity, identify high burden areas, and evaluate the impact of control interventions [21–25]. However, TPR is subject to bias due to changes in the incidence of non-malaria fevers and has a complex, non-linear relationship with malaria incidence [5, 7]. In addition, given that this metric is expressed as a proportion, it is commonly used as a qualitative measure as it is difficult to translate changes in TPR into meaningful quantitative measures needed to allocate resources and assess impact. In this study from 5 highly endemic areas of Uganda, temporal changes in TPR correlated poorly with changes in malaria incidence, with small changes associated with large changes in incidence. This is not surprising as when the burden of malaria is very high, TPRs can become nearly “saturated” well before malaria incidence has peaked. In a study from 15 villages in Western Uganda, the relationship between village level estimates of TPR and malaria incidence was best represented by an exponential model [6]. In this study, the correlation between TPR and malaria incidence was poor at low transmission levels, with large changes in TPR associated with minimal changes in malaria incidence. The correlation improved among villages with higher transmission intensity where the TPRs ranged from 10–50%. However, this study did not address the other end of the spectrum when transmission intensity becomes very high and TPRs exceed 50%, as was observed in a majority of the time points for all 5 sites included in this report. Taken together, these data suggest that in Uganda TPR and malaria incidence have a non-linear relationship and correlate poorly when transmission is either relatively low or relatively high. In contrast to these data from Uganda, in a study from Yunnan Province of China annual estimates of TPR and malaria incidence had a strong linear relationship with an adjusted R^2^ value of 0.85 [26]. In this study, malaria burden changed dramatically with annual TPRs declining from a high of 13% to less than 1% and malaria incidence declining from a high of 648 to 23 cases per 100,000 person years.

TCM, defined as the total laboratory confirmed cases of malaria per unit time, has also been used as a surrogate measure of malaria incidence. TCM is simple to measure, and unlike TPR, is quantitatively easy to interpret and not constrained by an upper limit. However, TCM is directly dependent on access to care and diagnositc testing and therefore highly susceptible to bias by these factors. For example, in a study from the Democratic Republic of the Congo evaluating trends in reported malaria cases between 2005 and 2014, a sharp increase in confirmed cases after 2010 was presumed to be due to the introduction and scale up in RDTs rather than a true increase in the incidence of malaria [27]. The study presented in this report benefited from an enhanced surveillance system where almost all patients with suspected malaria underwent diagnostic testing using an RDT. Indeed, in this study with limited potential source of bias acruing from access to care and diagnositc testing, temporal changes in TCM tracked much better with changes in malaria incidence compared to temporal changes in TPR. In addition, site-specific temporal changes in TCM had a strong linear relationship with malaria incidence, meaning that within an individual health facility relative changes in TCM and malaria incidence were proportionate (e.g. a 75% increase in TCM would be associated with 3 times the increase in malaria incidence compared to a 25% increase in TCM). However, because the slopes of the linear relationships between TCM and malaria incidence varied from site to site, changes in TCM could not be directly translated into changes in malaria incidence (i.e. a 50% in TCM did not necessarily correspond with a 50% increase in malaria incidence). This is not surprising given that TCM is highly dependent on the number of patients who access a health facility, which can vary from site to site.

This study had several limitations. First, estimates of malaria incidence came from catchment areas around each MRC and could have been associated with inaccuracies in the numerator (cases of malaria per unit time) and/or the denominator (population at risk). It was assumed that all cases of malaria within the catchment areas were captured at their respective health facilities, which could have led to an underestimation of the true incidence of malaria. Population denominators came from publicly available datasets which utilized available census data and satellite imagery for mapping settlements [28]. Errors in population estimates could have led to either an overestimation or underestimation of the true incidence of malaria. However, it is likely that potential bias in estimating malaria incidence was non-differential with respect to calendar time and, therefore, should not have had a significant impact on the analyses performed. Second, measurements of TPR and TCM were derived from all patients who presented to the MRCs while estimates of malaria incidence were derived only from the subset of patients who resided in the catchment areas around the MRCs. Differences between patients who did and did not reside in the catchment areas could have influenced the study findings, although in a previous study from Uganda adjustment for area of residence did not influence temporal trends in TPR [29]. Third, this study was conducted at health facilities that were part of an enhanced malaria surveillance network where support was provided to maximize the use of laboratory testing and prevent stock-outs of essential commodities. Thus, care should be taken when generalizing results to other settings were the reporting of laboratory confirmed malaria may be affected by poor malaria case management. Finally, this study only included data from areas of Uganda with high transmission intensity and should not be generalized to lower transmission settings.

## Conclusion

Conducting high quality malaria surveillance in high transmission settings is critical, as these areas disproportionately contribute to malaria morbidity and should be prioritized for control interventions. High burden areas represent a unique challege as large changes in disease incidence may go unnoticed or underappreciated. In this study, a relatively novel approach was used to estimate malaria incidence using routinely collected data and identifying catchment areas around health facilities. Temporal changes in TPR correlated poorly with changes in malaria incidence and did not provide a very useful metric for monitoring trends in disease burden. In contrast, TCM in a setting where laboratory testing for malaria was almost universal was strongly predictive of relative changes in malaria incidence over time at individual health facilities. However, TCM alone cannot be used to estimate malaria incidence or quantify changes in malaria incidence. There should be a continued emphasis on improving the quality of health facility-based malaria surveillance and maximizing the utility of these data through improved metrics and an understanding of their characteristics.

## Supplementary Information

**Additional file 1: Appendix 1.** Data captured on the HMIS 031 standardized form.

**Additional file 2: Appendix 2.** Maps of villages and parishes surrounding each MRC. Catchment area around each MRC used to estimate malaria incidence surrounded by black border.

## Data Availability

The datasets used for this study are available from the corresponding author on reasonable request.

## Abbreviations

HMIS: Health Management Information Systems
MRCs: Malaria Reference Centres
NMCD: National Malaria Control Division
TPR: Test positivity rate
TCM: Total laboratory confirmed cases of malaria
UMSP: Uganda Malaria Surveillance Project
WHO: World Health Organization
