# Supplementary material for: Relationships between test positivity rate, total laboratory confirmed cases of malaria, and malaria incidence in high burden settings of Uganda: an ecological analysis
Source: Malar J. 2021 Jan 13;20:42. doi: 10.1186/s12936-021-03584-7 (PMC7805073; doi:10.1186/s12936-021-03584-7)
Supplement: Supplementary file 2 — Additional file 2: Appendix 2. Maps of villages and parishes surrounding each MRC. Catchment area around each MRC used to estimate malaria incidence surrounded by black border. [file 12936_2021_3584_MOESM2_ESM.pdf]

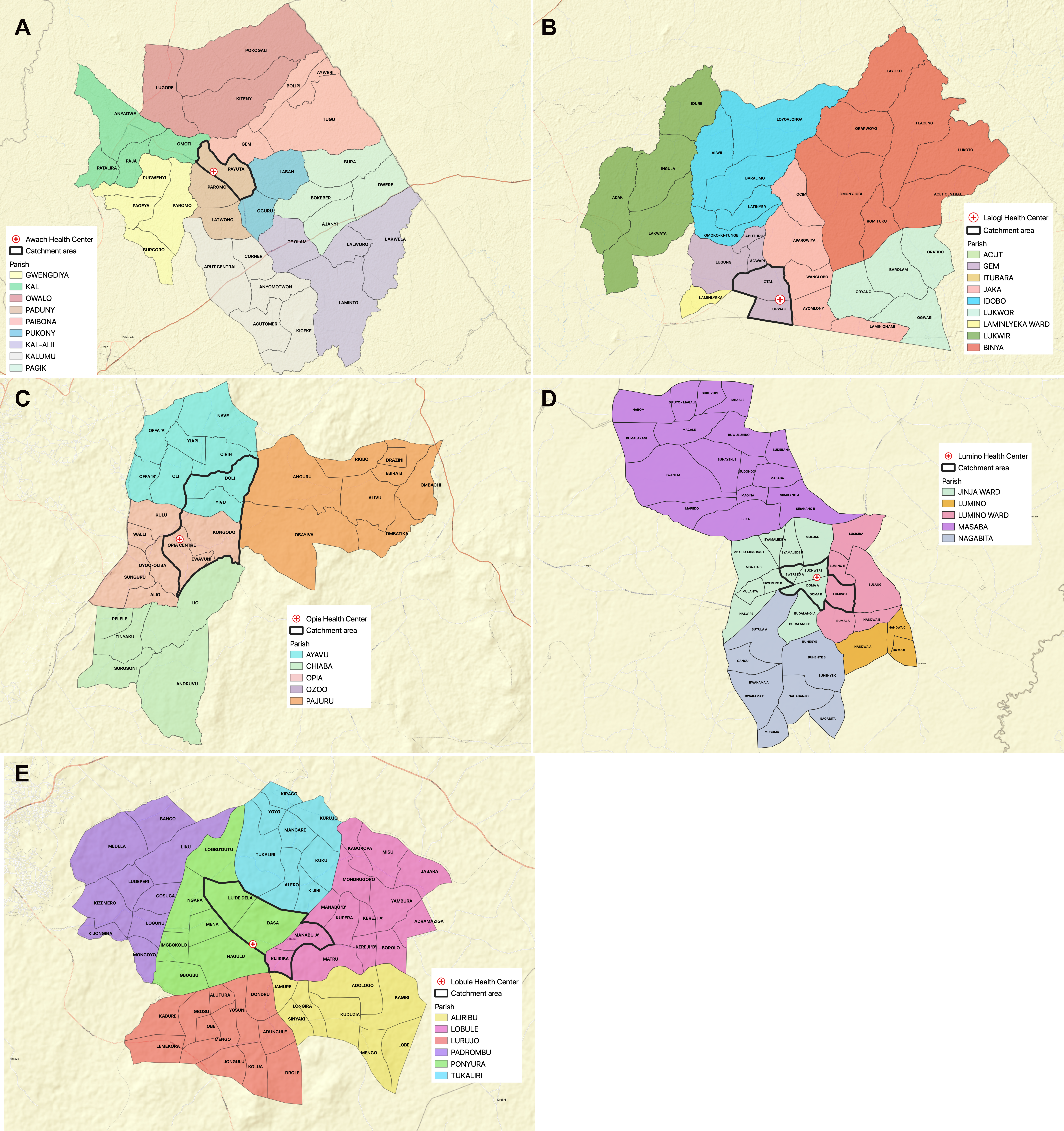
A
POKOGALI
LUGORE
KITENY
BOLIPII
AYWERI
TUGU
ANYADWE
OMOTI
GEM
PAYUTA
PATALIRA
PAJA
PUGWENYI
PAROMO
LABAN
BURA
DWERE
PAGEYA
PAROMO
BOKEBER
OGURU
LATWONG
AJANYI
TE OLAM
LALWORO
LAKWELA
BURCORO
CORNER
ARUT CENTRAL
ANYOMOTWON
LAMINTO
ACUTOMER
KICEKE
Awach Health Center
Catchment area
Parish
GWENGDIYA
KAL
OWALO
PADUNY
PAIBONA
PUKONY
KAL-ALII
KALUMU
PAGIK
B
LAYOKO
IDURE
LOYOAJONGA
TEACENG
ORAPWOYO
LUKOTO
ALWII
INGULA
BARALIMO
OCIM
OMUNYJUBI
ACET CENTRAL
ADAK
LATINYER
ROMITUKU
LAKWAYA
OMOKO-KI-TUNGE
ABUTURU
APAROWIYA
ORATIDO
AGWARI
LUGUNG
BAROLAM
OTAL
WANGLOBO
ORYANG
LAMINLYEKA
OPWAC
AYOMLONY
OGWARI
LAMIN ONAMI
Lalogi Health Center
Catchment area
Parish
ACUT
GEM
ITUBARA
JAKA
IDOBO
LUKWOR
LAMINLYEKA WARD
LUKWIR
BINYA
C
NAVE
OFFA 'A'
YIAPI
CIRIFI
OFFA 'B'
OLI
DOLI
YIVU
RIGBO
DRAZINI
EBIRA B
ANGURU
OMBACHI
ALIVU
KULU
KONGODO
WALLI
OPIA CENTRE
OBAYIVA
OMBATIKA
OYOO-OLIBA
SUNGURU
EWAVUNI
ALIO
LIO
PELELE
TINYAKU
SURUSONI
ANDRUVU
Opia Health Center
Catchment area
Parish
AYAVU
CHIABA
OPIA
OZOO
PAJURU
D
HABOMI
SIFUYO - MAGALE
BUKUYUDI
MBAALE
BUMALAKANI
MAGALE
BUWULUHIRO
BUDEBANI
BUHAYENJE
LWANIHA
MUDONDO
MASABA
MAGINA
SIRAKANO A
MAPEDO
SEKA
SIRAKANO B
LUSISIRA
SYAMALEDE A
MULUKO
MBAJJA MUGUNGU
SYAMALEDE B
MBAJJA B
BUCHWERE
LUMINO II
BWERERO A
BULANGI
BWERERO B
DOMA A
MULANYA
DOMA B
LUMINO I
NALWIRE
BUDALANGI A
BUTULA A
BUDALANGI B
SUMALA
NANDWA B
NANDWA C
BUHENYE
NANDWA A
BUYODI
GANGU
BUHENYE B
BUHENYE C
BWAKAMA A
BWAKAMA B
NAHABANJO
NAGABITA
MUSUMA
Lumino Health Center
Catchment area
Parish
JINJA WARD
LUMINO
LUMINO WARD
MASABA
NAGABITA
E
KIRAGO
YOYO
KURUJO
BANGO
MANGARE
MEDELA
LIKU
LOGBU'DUTU
TUKALIRI
KAGOROPA
MISU
KUKU
LUGEPERI
MONDRUGORO
JABARA
ALERO
KIJIRI
KIZEMERO
GOSUGA
NGARA
LU'DE'DELA
YAMBURA
MANABU 'B'
KUPERA
KEREJI 'A'
ADRAMAZIGA
LOGUNU
MENA
DASA
KIJONGINA
MANABU 'A'
IMGBOKOLO
KEREJI 'B'
BOROLO
MONGOYO
NAGULU
KIJIRIBA
MATRU
GBOGBU
JAMURE
ADOLOGO
ALUTURA
DONDRU
KAGIRI
KABURE
GBOSU
YOSUNI
LONGIRA
SINYAKI
KUDUZIA
OBE
ADUNGULE
LEMEKORA
MENGO
LOBE
JONGULU
MENGO
KOLUA
DROLE
Lobule Health Center
Catchment area
Parish
ALIRIBU
LOBULE
LURUJO
PADROMBU
PONYURA
TUKALIRI
