# Supplementary material for: Relationships between test positivity rate, total laboratory confirmed cases of malaria, and malaria incidence in high burden settings of Uganda: an ecological analysis
Source: Malar J. 2021 Jan 13;20:42. doi: 10.1186/s12936-021-03584-7 (PMC7805073; doi:10.1186/s12936-021-03584-7)
Supplement: Supplementary file 1 — Additional file 1: Appendix 1. Data captured on the HMIS 031 standardized form. [file 12936_2021_3584_MOESM1_ESM.pdf]

(1)	(2)	(3)	(4)	(5)	(6)	(7)	(8)	(9)			(10)	(11)	(12)
SERIAL NUMBER	NIN	NAME	AGE	SEX (M/F)	CLIENT CATEGORY (WRITE N/R/F)	RESIDENCE	NEXT OF KIN NAME AND CONTACT	NUTRITION ASSESSMENT			BLOOD PRESSURE (mmHg)	NEED FOR PALLIATIVE CARE	Tobacco Use
								MUAC (cm + (R-O/R-WO/Y/G)	BMI(SU/MU/MiU/N/OW/O)<br>BMI FOR AGE (SU/U/N/OW/O)	WEIGHT FOR HEIGHT Z SCORE (R-O/R-WO/Y/G)			
								WEIGHT (Kg)	WEIGHT FOR AGE Z SCORE (SU/U/N/OW/O)		BLOOD SUGAR (mm/l)		Tobacco Exposure
								HEIGHT/ LENGTH (cm)	HEIGHT/ LENGTH FOR AGE Z SCORE (N/S)				Alcohol use
	N I N	SURNAME		M/F	N/R/F	VILLAGE<br>PARISH	SURNAME<br>GIVEN NAME	cm (R-O/R-WO/Y/G)	SU/MU/MiU/N/OW/O<br>(SU/U/N/OW/O)	z-score (R-O/R-WO/Y/G)	mmHg	(YES/NO)	TOBACCO USE
		GIVEN NAME				SUBCOUNTY	PHONE CONTACT	Kg	Z-Score (SU/U/N/OW/O)		mm/l		TOBACCO EXPOSURE
		PHONE CONTACT				DISTRICT	RELATIONSHIP	cm	N/S				ALCOHOL USE

(13)			(14)				(15)		(16)	(17)	(18)	(19)	(20)
MALARIA TEST			TB				TICK CLASSIFICATION		DIAGNOSIS	PRESCRIPTION	DISABILITY*(Write code of disability)*	REF. IN (Yes/No)	REF.OUT (Yes/No)
FEVER (YES/ NO)	TESTS DONE (B/S, RDT/ND)	RESULTS (POS/NEG /NA)	PRESUMED TB CASE (Y/N)	PATIENT SENT TO THE LAB (Y/N)	LAB. TB RESULT (POS/ NEG/ NA)	LINKED TO TB CLINIC (Y/N)	NEW - ATTENDANCE	RE - ATTENDANCE					
(YES/ NO)	(B/S, RDT/ND)	(POS/NEG /NA)	(Y/N)	(Y/N)	(POS/ NEG/ NA)	(Y/N)<br>Where<br>Unit TB No.						(Yes/No)	(Yes/No)
